# Visualisation of a flexible modular structure of the ER folding-sensor enzyme UGGT

**DOI:** 10.1038/s41598-017-12283-w

**Published:** 2017-09-22

**Authors:** Tadashi Satoh, Chihong Song, Tong Zhu, Takayasu Toshimori, Kazuyoshi Murata, Yugo Hayashi, Hironari Kamikubo, Takayuki Uchihashi, Koichi Kato

**Affiliations:** 1 Graduate School of Pharmaceutical Sciences, Nagoya City University, 3-1 Tanabe-dori, Mizuho-ku, Nagoya 467-8603 Japan; 2 JST, PRESTO, 3-1 Tanabe-dori, Mizuho-ku, Nagoya 467-8603 Japan; 3 National Institute for Physiological Sciences, 5-1 Higashiyama, Myodaiji, Okazaki, Aichi 444-8787 Japan; 4 Okazaki Institute for Integrative Bioscience, 5-1 Higashiyama, Myodaiji, Okazaki, Aichi 444-8787 Japan; 5 Institute for Molecular Science, National Institutes of Natural Sciences, 5-1 Higashiyama, Myodaiji, Okazaki, Aichi 444-8787 Japan; 6 School of Physical Sciences, 5-1 Higashiyama, Myodaiji, Okazaki, Aichi 444-8787 Japan; 7 School of Life Science, SOKENDAI (The Graduate University for Advanced Studies), 5-1 Higashiyama, Myodaiji, Okazaki, Aichi 444-8787 Japan; 8 Graduate School of Materials Science, Nara Institute of Science and Technology, 8916-5, Takayama, Ikoma, Nara, 630-0192 Japan; 9 Department of Physics, Nagoya University, Furo-cho, Chikusa-ku, Nagoya 464-8602 Japan

## Abstract

In the endoplasmic reticulum (ER), a protein quality control system facilitates the efficient folding of newly synthesised proteins. In this system, a series of *N*-linked glycan intermediates displayed on the protein surface serve as quality tags. The ER folding-sensor enzyme UDP-glucose:glycoprotein glucosyltransferase (UGGT) acts as a gatekeeper in the ER quality control system by specifically catalysing monoglucosylation onto incompletely folded glycoproteins, thereby enabling them to interact with lectin–chaperone complexes. Here we characterise the dynamic structure of this enzyme. Our crystallographic data demonstrate that the sensor region is composed of four thioredoxin-like domains followed by a β-rich domain, which are arranged into a C-shaped structure with a large central cavity, while the C-terminal catalytic domain undergoes a ligand-dependent conformational alteration. Furthermore, small-angle X-ray scattering, cryo-electron microscopy and high-speed atomic force microscopy have demonstrated that UGGT has a flexible modular structure in which the smaller catalytic domain is tethered to the larger folding-sensor region with variable spatial arrangements. These findings provide structural insights into the working mechanism whereby UGGT operates as a folding-sensor against a variety of glycoprotein substrates through its flexible modular structure possessing extended hydrophobic surfaces for the recognition of unfolded substrates.

## Introduction

The endoplasmic reticulum (ER) possesses a sophisticated protein quality control system that ensures the appropriate folding and trafficking of newly synthesised proteins. In this system, *N*-linked oligosaccharides attached to the protein surface operate as tags for their quality control^1–4^. In the ER, *N*-linked glycan is initially introduced as a high-mannose-type tetradecasaccharide (Glc_3_Man_9_GlcNAc_2_). This sugar chain harbours three non-reducing terminal branches, the D1, D2 and D3 branches, among which the D1 branch is capped with three glucose residues. During glycoprotein maturation, the trimming of the first two glucose residues generates a critical ‘folding signal’, namely Glc_1_Man_9_GlcNAc_2_, which is specifically recognised by the lectins constituting chaperone complexes^3,5^. The chaperoning process is terminated by glucosidase II, which removes the innermost glucose residue, rendering the correctly folded glycoproteins ready to be transported to the Golgi complex for further glycan processing.

Remarkably, the quality control system is equipped with an elaborate backup mechanism employing a molecular ‘gatekeeper’ that can specifically catalyse reglucosylation against incompletely folded glycoproteins as potential substrates, thereby regenerating monoglucosylated glycoforms for their return to the chaperone-assisted folding process^3,5^. This gatekeeper function is executed by a unique enzyme, UDP-glucose:glycoprotein glucosyltransferase (UGGT)^6–10^. UGGT is a large Ca^2+^-dependent enzyme^11^ with a molecular mass of around 160 kDa, which has been putatively divided into two parts: a 125-kDa N-terminal region, which is thought to act as a folding-sensor region, and a C-terminal 35-kDa catalytic domain. Based on previous bioinformatic and crystallographic analyses, we proposed that the folding-sensor region harbours three tandem thioredoxin (Trx)-like domains followed by a β-strand-rich domain, displaying exposed hydrophobic patches putatively providing substrate-binding sites^12^. Although Trx domains are often found in the ER proteins involved in protein quality control^13^, the three-dimensional structural information regarding UGGT has thus far been available only for the isolated third Trx domain. Therefore, the structural basis of the working mechanism of this key enzyme remains to be elucidated. We herein characterise the overall structure of UGGT using integrative biophysical approaches and present the dynamic arrangement of its modular domains.

## Results and Discussion

### Crystal structures of the folding-sensor region and catalytic domain of UGGT

**Figure 1:**
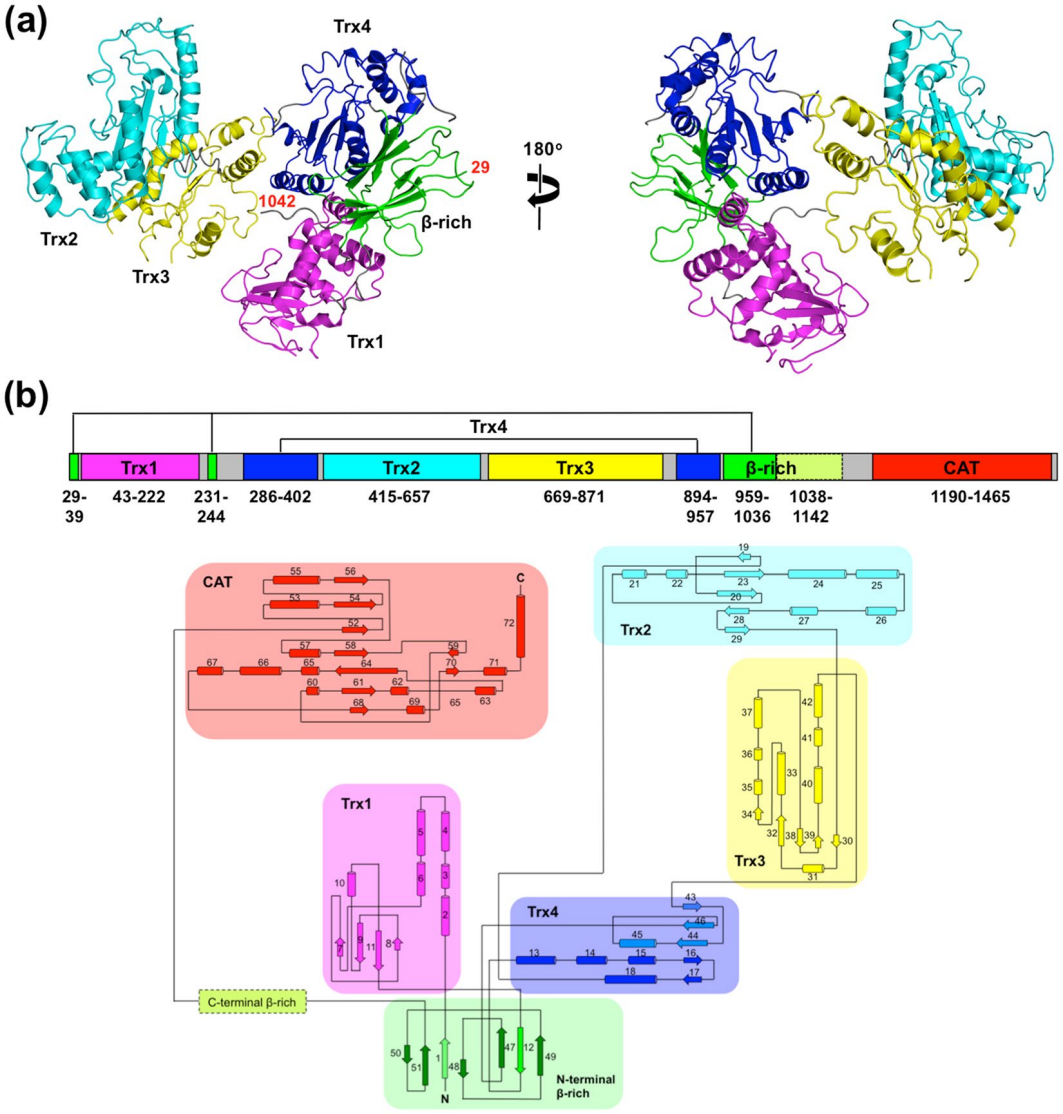
Crystal structure of N-terminal folding-sensor region of UGGT. (**a**) Crystal structure of UGGT^N^ is shown as ribbon models with indication of the N- and C-terminal positions with the residue numbers. Trx1, Trx2, Trx3, Trx4 and the N-terminal part of the β-rich domain are coloured magenta, cyan, yellow, blue and green, respectively. (**b**) Domain structure and topology diagram of *T. dupontii* UGGT.

We determined the crystal structure of the N-terminal folding-sensor region of UGGT (UGGT^N^, residues 29–1142) at a 3.1-Å resolution (Fig. 1a and Supplemental Tables S1–3). Beyond expectations based on our previous bioinformatic analysis^12^, the current crystallographic data revealed that UGGT^N^ was composed of four Trx-like domains (designated as Trx1–4), followed by a β-rich domain with a unique topology (Fig. 1b). The overall structure of UGGT^N^ displayed a C-shaped structure containing a large central cavity with 60 × 80 × 120 Å dimensions. The N-terminal Trx1 domain showed a unique Trx-like fold in which the four-helix subdomain was not inserted between β3 and β4 but preceded the α1-helix (Supplemental Fig. S1a). In contrast, Trx2 and Trx3 domains exhibited a typical Trx-like fold comprising a four- or three-stranded β-sheet surrounded by six α-helices, in which the four-helix subdomain was inserted between β3 and β4 (Supplemental Fig. S1b,c). The crystal structure of the Trx3 domain from *Thermomyces dupontii* was essentially identical to the isolated domain structure from *Chaetomium thermophilum*, which, however, indicated that the C-terminal α6-helix was disordered in the presence of a detergent molecule as a crystallising agent occupying a potential substrate-binding hydrophobic surface^12^. In the present *T. dupontii* UGGT^N^ structure, the α6-helix was ordered in the detergent-free condition. The Trx4 domain represented a typical Trx-like fold comprising a four-stranded β-sheet surrounded by six α-helices, which, however, exhibited an unusual topology (Supplemental Fig. S1d). Namely, in the four-helix subdomain, one core α-helix and β-strand are encoded by the N-terminal segment (residues 275–410), while three core β-strands and α-helix are encoded by the C-terminal segment (residues 897–950) (Fig. 1b). Such uncommon domain architecture was also observed in the β-rich domain comprising six-stranded β-strands (Supplemental Fig. S1e), which are encoded by three discrete segments (residues 29–39, 231–244 and 959–1036) (Fig. 1b). Although we hypothesised that UGGT possesses a β-strand-rich domain comprising residues 942–1149 based on the bioinformatic analysis^12^, only the N-terminal subdomain comprising residues 959–1036 gave unambiguous electron density (Supplemental Fig. S1e and f), suggesting motional freedom in this domain. In the central cavity of the UGGT^N^ C-shaped structure, remarkable hydrophobic patches were found (Supplemental Fig. S2), suggesting their possible involvement in folding-sensor function.

**Figure 2:**
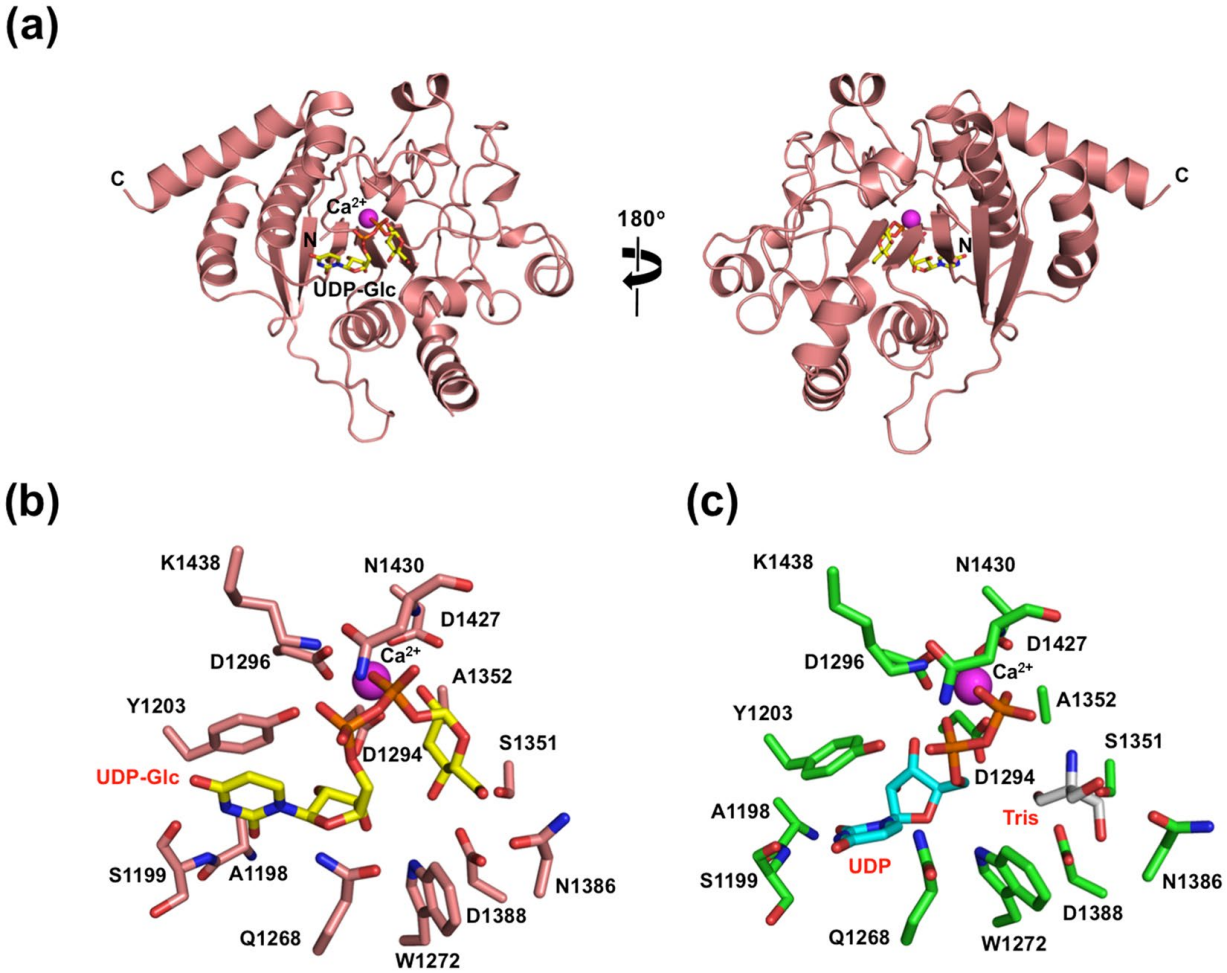
Crystal structure of C-terminal catalytic domain of UGGT. (**a**) Crystal structure of CAT domain in complex with UDP-Glc is shown as ribbon models. The bound UDP-Glc and Ca^2+^ are shown as stick and sphere models, respectively. The catalytic active site of UGGT in the UDP-Glc or UDP-bound form is shown in (**b**) and (**c**), respectively. Residues involved in binding to Ca^2+^ and UDP-Glc or UDP are numbered and shown in the stick model. The UDP-Glc and UDP molecules are coloured yellow and cyan.

We determined the crystal structure at a 1.40-Å resolution of the C-terminal catalytic domain (CAT, residues 1190–1480) in complex with a UDP-glucose donor substrate and Ca^2+^ (Fig. 2a and Supplemental Table S4), which are required for the enzymatic activity^3^. In the CAZy classification, the CAT domain belongs to the GT24 family, which is supposed to share structural similarity with the GT8 family^14^. This was confirmed by the present crystal structure showing a GT-A fold comprising nine β-strands and 12 α-helices. The UDP-glucose and Ca^2+^ ligands were accommodated in the active site containing a DXD motif (D1294-A1295-D1296) (Fig. 2b), in which the ligand binding residues are essentially identical across species together with the retaining GT8 glycosyltransferases^14^ (Supplemental Fig. S3), suggesting that the enzymatic mechanism of UGGT is evolutionarily conserved. Furthermore, we determined the crystal structure of the CAT domain in complex with UDP and Ca^2+^ at a 1.35-Å resolution (Fig. 2c, Supplemental Fig. S4 and Supplemental Table S4). In the crystal structure, the Tris molecule was accommodated in the corresponding binding site on the glucose moiety of UDP-glucose. In comparison with UDP-glucose-bound complex, 1379–1387 and 1427–1438 loops containing Asn1386 and Asn1430 located closer to the glucose- or Tris-binding site undergo significant structural changes upon UDP binding (Supplemental Fig. S4b), suggesting its mechanism of release of the monoglucosylated product. In both crystal structures, a remarkable hydrophobic patch was found around the active site (Supplemental Fig. S5), implying its possible contribution to the folding-sensing mechanism of the catalytic domain. An affinity labelling experiment suggested that the catalytic domain is involved in interaction with a hydrophobic aglycon^15^.

### Overall structure of UGGT

**Figure 3:**
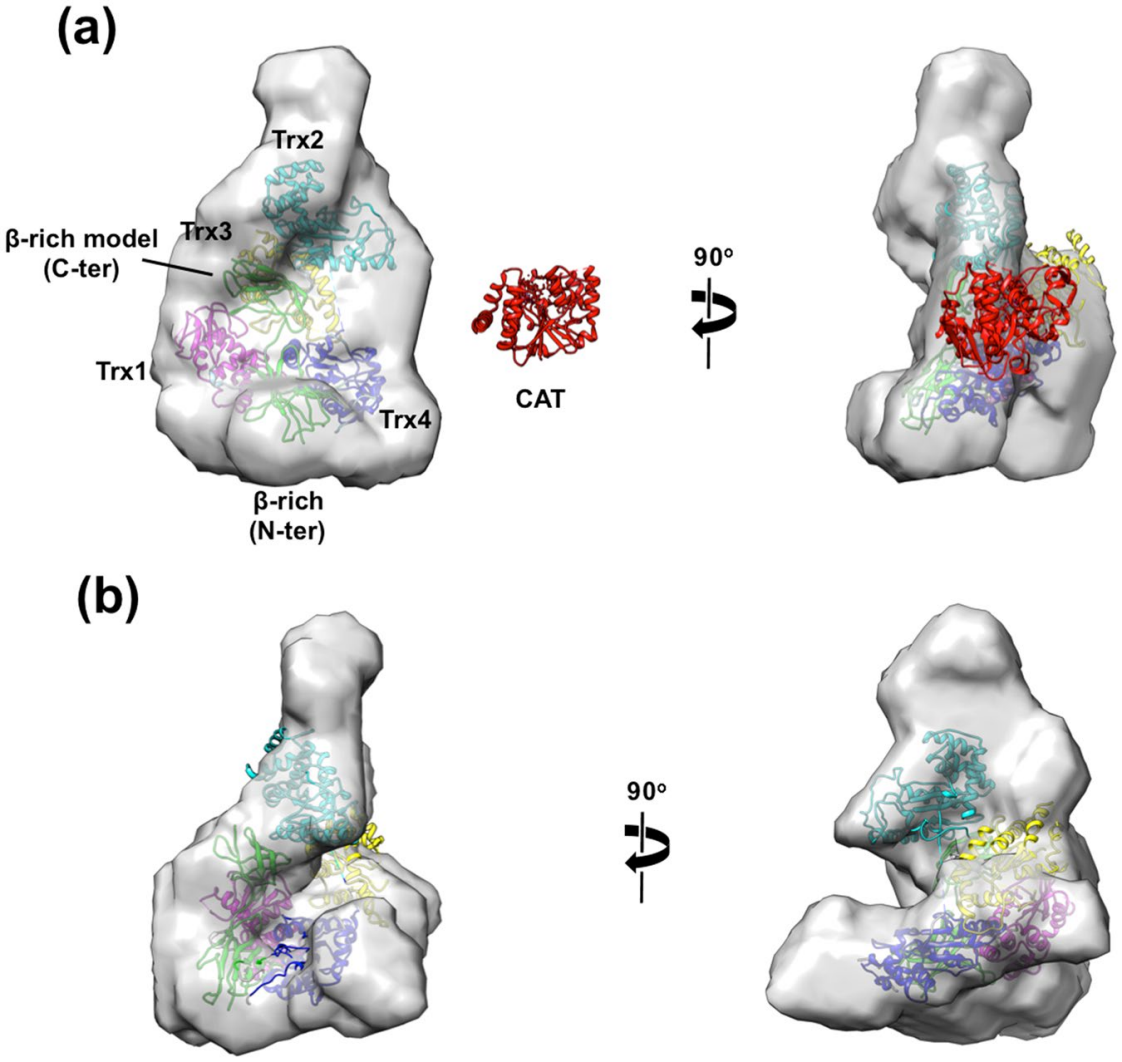
SAXS-derived structural model of full-length and folding-sensor domain of UGGT. *Ab initio* shape models of UGGT^FL^ and UGGT^N^ are presented in (**a**) and (**b**), respectively. The crystal structure of UGGT^N^ fitted into the shape modes is coloured as in Fig. 1. Homology model of putative C-terminal β-rich domain created by SWISS-MODEL^34^ is placed at the region where the partial electron density was observed in the UGGT^N^ crystal structure. The crystal structure of the CAT domain is shown on the same scale as a reference.

To obtain information on its overall structure, we performed small-angle X-ray scattering (SAXS) experiments of full-length UGGT (UGGT^FL^) together with UGGT^N^ in solution. The radius of gyration (*R*g), the maximum dimension (*D*_max_) and the apparent molecular mass were estimated to be 45 ± 1 Å, 150 Å and 164 ± 6 kDa for UGGT^FL^ and 47 ± 1 Å, 155 Å and 146 ± 2.3 kDa for UGGT^N^, respectively. These data indicate their monomeric structures in solution (Supplemental Figs S6,S7 and Table S3). However, the estimated *R*g and *D*_max_ values of UGGT^N^ were significantly different from those estimated from the crystal structure (Fig. 1a), that is, approximately 36.4 and 120 Å, respectively, although the crystal structure contained several disordered segments including the C-terminal part of the β-rich domain. Based on the SAXS data, we constructed three-dimensional shape models of UGGT^FL^ and UGGT^N^ illustrating the anisotropic elongated C-shaped structures (Fig. 3). The UGGT^N^ crystal structure nicely fitted into the shape models. Although comparison between the UGGT^FL^ and UGGT^N^ models indicated that the CAT domain is located in the space surrounded by the Trx2, Trx3 and Trx4 domains in the UGGT^N^ model, the crystal structure of the CAT domain could not be adequately fitted with the putative site in terms of the matching of volume density (Fig. 3a), suggesting that the CAT domain is considerably mobile in solution. Besides the catalytic domain, an extra protrusion was found around the Trx2 domain, suggesting its mobility (Fig. 3a,b). Taken together, these results suggest that UGGT has significant conformational heterogeneity because of the flexible nature of the Trx2 and CAT domains in solution.

**Figure 4:**
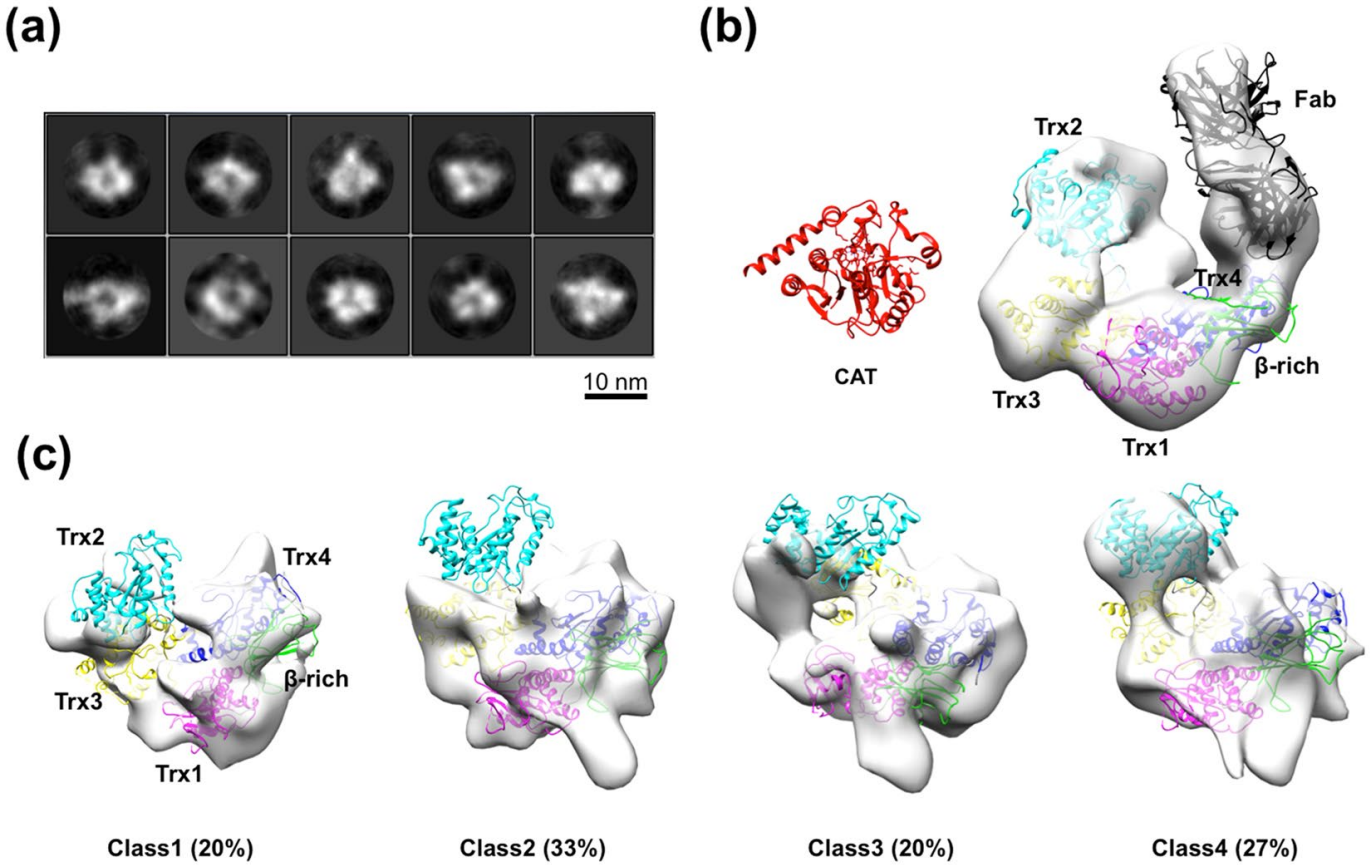
Cryo-EM structure of full-length UGGT. (**a**) 2D classes of UGGT particle subjected to single-particle cryo-EM analysis. (**b**) Negative-stain EM 3D reconstruction of UGGT with the Fab of monoclonal antibody directed against the Trx4 domain. The crystal structure of UGGT^N^ fitted into the negative-stain EM map is coloured as in Fig. 1. The crystal structure of the CAT domain is shown on the same scale as a reference. The Fab fragment of the anti-Trx4 antibody is coloured black. (**c**) 3D classification of cryo-EM structures of UGGT. Each structure showed significant variations in the cryo-EM maps, but commonly shared a large central cavity.

To collect conformational snapshots of UGGT, we performed electron microscopy (EM) of UGGT^FL^. Approximately 18,000 particles were picked from the 297 cryo-EM images and subjected to 2D classification. Intriguingly, the obtained 2D class averages showed significant particle heterogeneity (Fig. 4a). The 2D class data were sorted into four 3D classes with almost equal populations (20–33%), indicating the structural variation of UGGT. Owing to the conformational heterogeneity and the consequent limited-resolution images, the crystal structure of UGGT^N^ could not be unambiguously fitted into the EM maps. Therefore, we performed domain mapping using a monoclonal antibody directed against the UGGT segment 29–468 corresponding to parts of β-rich, Trx1, or Trx4 domain (Supplemental Fig. S8). Based on the negative-stain EM data of UGGT^FL^ complexed with the Fab fragment of this antibody, we successfully identified the respective domains in the EM map. Concomitantly, the EM data together with dot blot analysis showed that the UGGT antibody specifically recognises the Trx4 domain. Similar to the UGGT^FL^ crystal structure, the Trx4 domain is located at one edge of the C-shaped structure in the EM image (Fig. 4b and Supplemental Fig. S9). The negative-stain EM data demonstrated that UGGT^FL^ exhibits a cradle-like C-shaped structure with a central cavity. Whereas most of the individual parts of UGGT^N^ crystal structures could be nicely fitted into the EM map, the part of the β-rich domain disordered in the crystal structure was still not observed regardless of the EM images of the intact protein. An extra density map was observed at the cavity surrounded by Trx1–Trx3 domains, indicating that the CAT domain would be located there (Fig. 4b). However, the crystal structure of the CAT domain did not perfectly match the extra EM map volume. Consequently, these negative-stain EM data suggest that the C-terminal part of the β-rich and CAT domains as well as the linker connecting them are quite flexible and therefore invisible. Using information on the domain mapping, the UGGT^N^ crystal structure can be fitted into the four classes of the cryo-EM maps (Fig. 4c), confirming that the UGGT^FL^ possesses a large central cavity. In the cryo-EM images, the Trx1 and Trx4 as well as the N-terminal part of β-rich domains, which were tightly associated with each other in the crystal structure (Fig. 1a), were observed with variable spatial arrangements, whereas the Trx2 and Trx3 domains were more extensively dislocated, precluding unambiguous mapping of the Trx2 domain in these EM images. Similar to the negative-stain EM data (Fig. 4b), the cryo-EM data provide no interpretable density corresponding to the CAT domain in the EM map (Fig. 4c). On the basis of the EM data together with the SAXS, we concluded that UGGT possesses a high degree of motional freedom of the CAT domain with respect to the folding-sensor region, which also exhibits significant conformational variability due to the conformational dynamics of its modular structure.

### Visualisation of the flexible modular structure of UGGT

**Figure 5:**
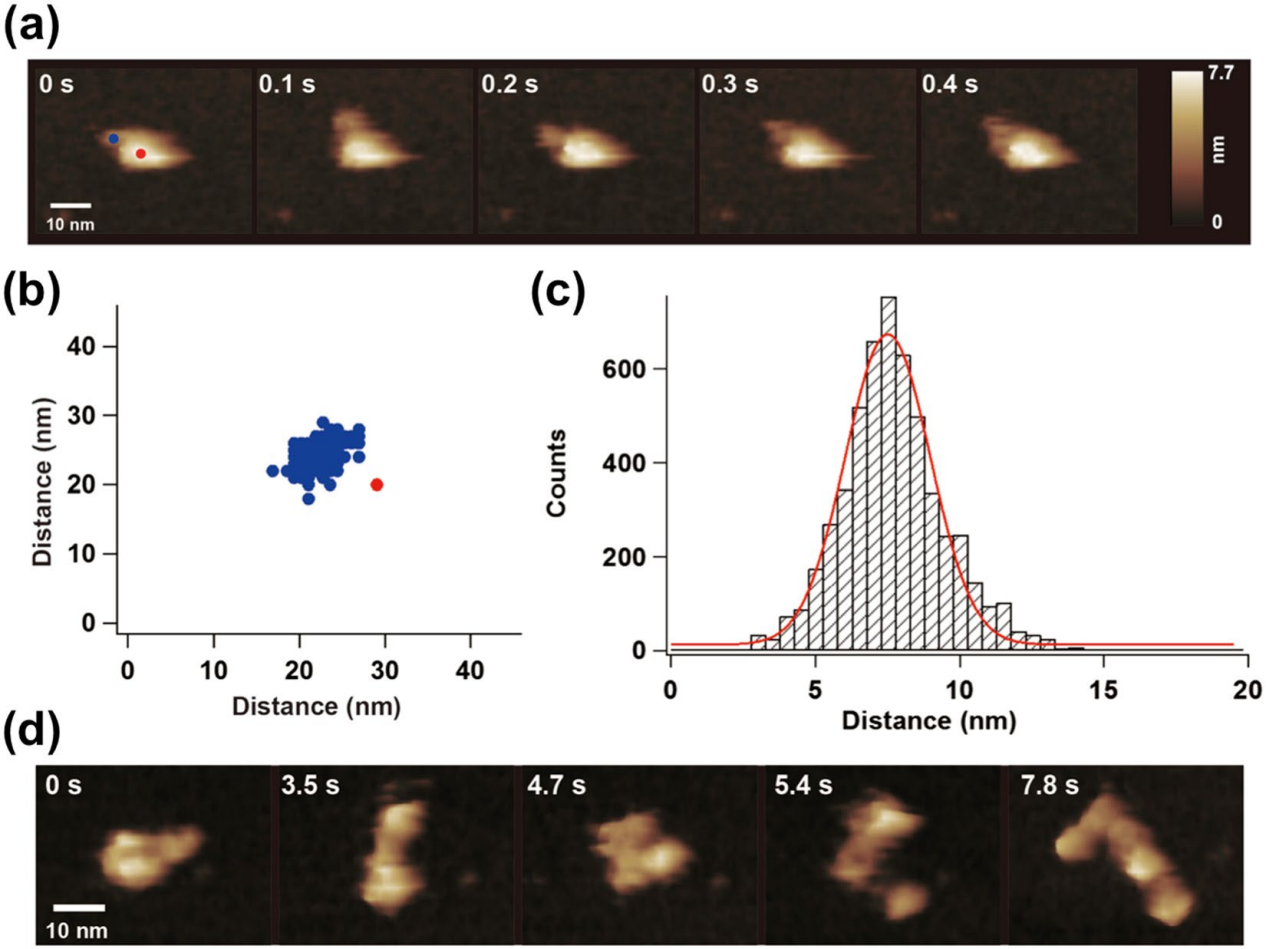
HS-AFM structure of full-length UGGT. (**a**) Typical HS-AFM image of N-terminally His_6_-tagged UGGT^FL^ (Sample #10, Supplemental Video S1). (**b**) Distribution of centre positions of the C-lobes (blue circles) relative to the centre of the N-lobes of UGGT measured from the HS-AFM movie. (**c**) Histogram and fitting of the normal distribution curve for the distance between the centres of two lobes of UGGT in the HS-AFM real-time images (*n = *5374, Samples #1–10, Supplemental Fig. S5). (**d**) Typical HS-AFM image of a deformed UGGT^FL^ (N-terminally His_6_-tagged construct, Supplemental Video S3).

We performed high-speed atomic force microscopy (HS-AFM) to characterise the dynamic nature of UGGT further. The AFM images of UGGT exhibited one larger lobe and one smaller one with their dimensions concordant with SAXS, cryo-EM and crystallographic data (Supplemental Fig. S10). When the N-terminally His_6_-tagged UGGT was immobilised onto a Ni^2+^-treated mica surface, the smaller lobe dynamically moved around the larger lobe, the latter being the least mobile (Fig. 5a and Supplemental Video S1). In contrast, when the C-terminally tagged construct was used, the position of the larger lobe dynamically fluctuated (Supplemental Video S2). These results clearly indicate that the observed larger and smaller lobes of UGGT correspond to the N-terminal folding-sensor region and the C-terminal catalytic domain, respectively. These observations were consistent with the bioinformatic-based prediction that the N- and C-lobes are connected via an unstructured linker (Fig. 1b)^12^. In the HS-AFM image, the relative positions of the N- and C-lobes were thus variable but not randomly distributed (Fig. 5b and Supplemental Fig. S11). The most frequently observed distances between the centres of two lobes were estimated to be 75 Å (Fig. 5c). This distance value was comparable to previous biochemical data indicating that UGGT can transfer glucose to N-glycans positioned at least 40 Å from the unstructured regions^7^.

Upon the intentional deformation of immobilised UGGT using the AFM tip, the N-lobe was disrupted into at least four domains (Fig. 5d and Supplemental Video S3), consistent with the crystallographic data suggesting that UGGT^N^ consists of the four Trx-like domains tightly associated with the β-rich domain (Fig. 1a). After removal of the force applied using the AFM tip, the disrupted N-lobe spontaneously recovered to the large globular particle, indicating the reversible nature of the domain assembly of the N-lobe (Supplemental Video S3).

## Conclusion

Here, we characterised the overall structure and dynamic property of UGGT using an integrative approach by combining X-ray crystallography, SAXS, cryo-EM and HS-AFM. Our results reveal that UGGT has a modular structure in which the 35-kDa catalytic domain is tethered to the 125-kDa folding-sensor region. The N-terminal folding-sensor region is composed of the four Trx-like domains and the β-domain assembled into the C-shaped structure with structural plasticity harbouring the central cavity displaying hydrophobic patches, which putatively accommodates incompletely folded glycoproteins. These findings provide structural insights into the working mechanism whereby UGGT operates as a folding-sensor against a variety of glycoprotein substrates through its flexible modular structure possessing the extended hydrophobic surfaces for the recognition of unfolded substrates.

## Methods

### Protein expression and purification

The artificial codon-optimised UGGT^FL^ gene (residues 29–1480) from *T. dupontii*, a thermophilic fungus, was designed using the genomic DNA database (Talth1p4_002475, http://genome.fungalgenomics.ca) and purchased from Genscript (Japan). The UGGT^FL^ and N-terminal folding-sensor region (UGGT^N^, residues 29–1142) genes were subcloned into the *Bam*HI and *Sal*I sites of a modified pCold-GST vector^12^. Expression plasmids containing N- or C-terminally His_6_-tagged UGGT^FL^ genes were constructed using the inverse PCR method with KOD plus DNA polymerase (TOYOBO). Domain-specific Met-labelled UGGT^N^ mutants were created by L36M/I89M/V118M (β-Trx1*), L313M/L355M/I383M (Trx4*), I448M/L752M/L808M (Trx2-3*) and I931M/L966M/I1038M (Trx4*-β) mutations for protein chain tracing of UGGT^N^ crystal structure determination. A series of UGGT^N^ fragments were prepared for epitope mapping of monoclonal antibodies directed against UGGT (see Supplementary information, Fig. S8). The expression and purification of these recombinant proteins were performed in accordance with a protocol used for the crystallisation of the third Trx-like domain of *C. thermophilum* as previously described^12^. The resultant N- or C-terminally His_6_-tagged UGGT^FL^ proteins contained ‘GSHHHHHHGSHM’ or ‘HHHHHH’ sequences at the N- or C-terminus, respectively, after removing the GST tag.

The gene encoding the catalytic domain of UGGT (CAT, residues 1190–1480) was amplified by PCR and subcloned into the *Bam*HI and *Sal*I sites of a modified pCold-I vector (Takara Bio Inc.), in which the factor Xa site was replaced with the tobacco etch virus (TEV) protease recognition site. The CAT domain plasmid was introduced into *Escherichia coli* BL21-CodonPlus (DE3)-RIL (Agilent Technologies). The recombinant CAT domain was produced as an inclusion body in *E. coli* and subjected to oxidative refolding. The harvested cells were resuspended in buffer containing 50 mM Tris-HCl (pH 7.5) and 150 mM NaCl and lysed by sonication. The obtained inclusion bodies were extensively washed with the resuspension buffer containing 1 mM EDTA and 2% Triton X-100 and subsequently solubilised in 6 M guanidinium chloride, 50 mM Tris-HCl (pH 8.0) and 10 mM dithiothreitol. The solubilised protein was refolded by dilution (to a protein concentration of 0.2 mg/mL) in 50 mM Tris-HCl (pH 7.5), 10 mM CaCl_2_, 400 mM L-arginine, 5 mM reduced glutathione and 0.5 mM oxidised glutathione at 4 °C for 12 h. The unconcentrated refolded protein was dialysed against a large amount of buffer containing 20 mM Tris-HCl (pH 7.5), 2 mM CaCl_2_ and 150 mM NaCl to remove excess L-arginine. This buffer exchange procedure at low protein concentration was critical for the successful refolding of the CAT domain. After the dialysis, the refolded His_6_-tagged CAT domain was purified and concentrated using cOmplete His-Tag Purification Resin (Roche). Dialysis buffers containing 10 and 500 mM imidazole were used for column washing and as an elution buffer, respectively. Next, the His_6_-tag on the CAT domain was removed by TEV protease and dialysed against 20 mM Tris-HCl (pH 8.0) and 2 mM CaCl_2_. Finally, the untagged CAT domain was purified on a Resource Q anion exchange column (GE Healthcare) with a 0–0.5 M NaCl gradient.

### Crystallisation, X-ray data collection and structure determination

The SeMet-substituted CAT domain protein was concentrated to 4.0 mg/mL in 20 mM Tris-HCl (pH 8.0), 150 mM NaCl, 2 mM CaCl_2_ and 5 mM UDP-Glc and used for crystallisation. Optimised crystals were obtained by a hanging-drop vapour diffusion method in 24% PEG 3350 and 100 mM Tris-HCl (pH 9.0) at 20 °C after a few days. For X-ray diffraction data collection, the crystals were cryoprotected with a crystallisation buffer containing 24% PEG 3350, 100 mM Tris-HCl (pH 9.0), 2 mM CaCl_2_, 5 mM UDP-Glc and 15% glycerol and flash-cooled in liquid nitrogen. The UDP-Glc-bound CAT domain crystal was soaked into the crystallisation buffer containing an excess amount of UDP (10 mM), giving rise to the UDP-bound complex. For crystallisation of UGGT^N^ proteins, the SeMet-substituted UGGT^N^ was concentrated to 10.0 mg/mL in 20 mM Tris-HCl (pH 8.0), 150 mM NaCl and 2 mM CaCl_2_. The UGGT^N^ variants (β-Trx1*, Trx4*, Trx2–3* and Trx4-β*) were also dissolved and concentrated under the same conditions. Meanwhile, among the UGGT^N^ variants, a high-quality single crystal was not obtained only for the Trx4-β* mutant. The remaining UGGT^N^ proteins could all be successfully crystallised (under the conditions summarised in Tables S1–3). Complete data sets for the CAT domain and UGGT^N^ were collected, indexed, integrated and scaled using MOSFLM^16^, HKL2000^17^ and XDS^18^.

The crystals of the CAT domain complexed with UDP-Glc or UDP alone belonged to the space group *P*2_1_2_1_2_1_ with one molecule per asymmetric unit and diffracted up to resolutions of 1.40 and 1.35 Å, respectively. The crystal structure of UDP-Glc-bound CAT domain was solved by using the single-wavelength anomalous dispersion method with a crystal of the SeMet-substituted protein, while the UDP-bound form was determined by molecular replacement using the UDP-Glc-bound structure as a search model (regarding crystallographic parameters and refinement statistics, see Tables S1–S4). The initial phases were determined with the SHELX C/D/E programs^19^. The obtained electron density map was clear enough to be interpreted and the initial coordinates were built automatically using ARP/wARP^20^. The obtained UGGT^N^ crystals belonged to the apparent space group *P*6_2_22, and the structure was solved by using the single- and multi-wavelength anomalous dispersion method with SeMet- and Pt-labelled crystals, respectively (Supplemental Table S2). Based on the obtained electron density map in conjunction with the methionine marking approach, namely through guidance of anomalous signals from SeMet-labelled β-Trx1*, Trx2-3* and Trx4* crystals (Supplemental Table S2), the initial coordinates of UGGT^N^ were manually built. However, merohedral twinning of UGGT^N^ was suspected during refinement because the refined coordinates had an *R*_work_ of 32% and an *R*_free_ of 37% in the *P*6_2_22 space group, even in the proceeded stage for model building. Therefore, the diffraction data were processed with the lower-symmetry space group and the eventually twinning refinement in the *P*3_2_12 space group dramatically improved the refinement statistics, namely *R*_work_ */R*_free_ = 23.2%/27.8%. Manual model fitting to the electron density maps was performed using COOT^21^. The refinement procedure was performed with phenix.refine^22^ and REFMAC5^23^. The stereochemical quality of the final model was assessed using PROCHECK^24^. The molecular graphics were made using UCSF Chimera^25^ and PyMOL (http://www.pymol.org/).

### Small-angle X-ray scattering

The untagged forms of UGGT^FL^ and UGGT^N^ were used for the measurements of small-angle X-ray scattering (SAXS). The SAXS measurements were performed using the Nano-Viewer diffractometer system equipped with a MicroMax-007 X-ray generator (RIGAKU), with a Cu target (λ = 1.5418 Å) and PILATUS 200 K (DECTRIS). All samples were prepared in 10 mM Tris-HCl (pH 7.7) containing 150 mM NaCl and 2 mM CaCl_2_. The scattering profiles were collected from sample solutions at eight different concentrations ranging from 2 to 5 mg/mL at 23 °C. Ovalbumin (45 kDa; Sigma-Aldrich) was also measured and used as a standard protein to estimate the apparent molecular weight of UGGT. The exposure time was 30 min for each sample. The observed two-dimensional image data were circularly averaged and then the profile of the buffer solution was subtracted. The concentration dependence of the *R*g and the forward scattering intensity normalised by the weight per volume (mg/mL), *I*(0)/conc., were used for the SAXS analysis, in which the small-angle regions (0.0129–0.0285 Å^−1^ for UGGT^FL^ and 0.0129–0.0313 Å^−1^ for UGGT^N^) were subject to Guinier plotting (Supplemental Figs S6 and S7). The *P*(*r*) functions of UGGT^FL^ and UGGT^N^ were calculated from the SAXS profile extrapolated to a protein concentration of zero by using GNOM software^26,27^ (see Table S5 for the obtained parameters).

*Ab initio* shape modelling was performed using DAMMIN^28^ without structural restrictions such as point symmetry and particle anisometry. The 10 independently calculated models were averaged using DAMAVER^29^. Using the average model as a start model, we finally refined the shape model using DAMMIN. The refinement procedures were independently performed three times to check the reproducibility. The representative model is shown in Fig. 3a and b. The structural model of UGGT^N^ obtained from the crystallographic analysis was superposed into the shape models of UGGT^FL^ and UGGT^N^ by using SUPCOMB^29^.

### Electron microscopy

For the cryo-EM, untagged, UGGT^FL^ protein was used. An aliquot of 2.5 μL was applied on R1.2/1.3 holely carbon film on a molybdenum grid (Quantifoil Micro Tools GmbH) pre-treated by glow-discharging. The plunged-freezing of the specimen was performed with Vitrobot Mark-IV (FEI Company). The frozen grid was kept at liquid nitrogen temperature and loaded into a JEM2200FS electron microscope equipped with a 200-kV field emission electron source and an omega-type energy filter (JEOL Ltd.) using a Gatan 914 cryo-specimen holder (Gatan Inc.). A total of 297 images were collected on a DE20 direct detector camera (Direct Electron LP) at a detector magnification of 93,023 with an energy slit width of 20 eV using a low-dose mode. The image size was 0.69 Å per pixel on the camera. The images were processed by Relion 1.4 software^30^ after subjecting them to motion collection using the DE_process_frames.py script provided by the manufacturer. In Relion, the images were estimated the contrast transfer function after twice binning. Then, 33,195 particle images were collected from the 297 images using an implemented auto-picking programme. The particle images were subjected to 2D classification after sorting with cross-correlation coefficients. Three-dimensional structures were reconstructed from good 2D classes consisting of 17,762 particle images using initial models of UGGT generated from subtomogram averaging of electron tomography performed with the same data acquisition conditions as mentioned above. For domain mapping, specimens of untagged, UGGT^FL^ protein were reacted with an excess amount of the Fab fragment of UGGT-specific monoclonal antibody (regarding preparation and characterisation of this antibody, see Supplemental method and Fig. S8) for 2 hours at 4 °C. The antibody reacted specimens were applied onto lab-made carbon-coated EM grids glow-discharged beforehand. After removing excess solution with filter paper, the grids were stained with 2% uranyl acetate solution and dried after removing the staining with filter paper. The stained grids were observed and the images were collected and processed with the same data acquisition conditions and procedures as mentioned above.

### High-speed atomic microscopy

For HS-AFM of UGGT^FL^, N- or C-terminally His_6_-tagged UGGT proteins were used. We used laboratory-constructed, high-speed AFM apparatus^31^ with cantilevers (7-μm long, 2-μm wide and 90-nm thick) operated at room temperature. Typical values of the spring constant, resonant frequency and quality factor of the cantilever in aqueous solution were ~0.2 N/m, ~0.8 MHz and ~2, respectively. In the AFM imaging, the free and set-point oscillation amplitudes were approximately 1.5 nm and 90% of the former, respectively. The N- or C-terminally His_6_-tagged UGGT proteins were immobilised onto a Ni^2+^-treated AFM surface on which the histidine tagged to the molecules binds to the Ni^2+^ ions on the negatively charged mica surface in 10 mM Tris-HCl (pH 7.5), 150 mM NaCl and 2 mM CaCl_2_. The HS-AFM images were taken at a frame rate of 10 fps.

## Note

While this paper was under the peer review process, structural characterisation was reported for UGGT^FL^ from three different species, *Drosophila melanogaster* (fly), *Penicillium chrysogenum* (mesophilic fungus) and *Chaetomium thermophilum* (thermophilic fungus), by two independent research groups^32,33^. The SAXS and EM data of fly and mesophilic fungal UGGTs showed similar C-shaped structures with conformational heterogeneity, as observed in our thermophilic fungal UGGT. Regarding the other thermophilic fungal UGGT, crystal structures of UGGT^FL^ at a resolution of 2.8–4.3 Å with proteolytic cleavage at the linker connecting the N-terminal folding-sensor region and the CAT domain were determined, in addition to SAXS- and EM-derived structural models.

Coordinates and structural factors of our crystal structures of UGGT have been deposited in the PDB: UGGT^N^ (5Y7O) and CAT domain (UDP-Glc-bound form [5H18] and UDP-bound form [5Y7F]).

## Electronic supplementary material

Supplementary information

Supplemental Movie S1

Supplemental Movie S2

Supplemental Movie S3
