# Supplementary material for: Visualisation of a flexible modular structure of the ER folding-sensor enzyme UGGT
Source: Sci Rep. 2017 Sep 22;7:12142. doi: 10.1038/s41598-017-12283-w (PMC5610325; doi:10.1038/s41598-017-12283-w)
Supplement: Supplementary file 1 — Supplementary information [file 41598_2017_12283_MOESM1_ESM.pdf]

Supplementary information

Visualisation of a flexible modular structure of the ER folding-sensor enzyme UGGT

Tadashi Satoh[1,2,*], Chihong Song[3], Tong Zhu[1,4,5,6], Takayasu Toshimori[1,5], Kazuyoshi Murata[3,7], Yugo Hayashi[8], Hironari Kamikubo[8], Takayuki Uchihashi[9] and Koichi Kato[1,4,5,6*]

[1]Graduate School of Pharmaceutical Sciences, Nagoya City University, 3-1 Tanabe-dori, Mizuho-ku, Nagoya 467-8603, Japan; [2]JST, PRESTO, 3-1 Tanabe-dori, Mizuho-ku, Nagoya 467-8603, Japan; [3]National Institute for Physiological Sciences, [4]Okazaki Institute for Integrative Bioscience, and [5]Institute for Molecular Science, National Institutes of Natural Sciences, 5-1 Higashiyama, Myodaiji, Okazaki, Aichi 444-8787, Japan; [6]School of Physical Sciences and [7]School of Life Science, SOKENDAI (The Graduate University for Advanced Studies), 5-1 Higashiyama, Myodaiji, Okazaki, Aichi 444-8787, Japan; [8]Graduate School of Materials Science, Nara Institute of Science and Technology, 8916-5, Takayama, Ikoma, Nara, 630-0192, Japan; [9]Department of Physics, Nagoya University, Furo-cho, Chikusa-ku, Nagoya 464-8602, Japan

Supplemental methods

Epitope mapping of anti-UGGT antibody

We purchased anti-UGGT antibody, which was created by a phage display technique using $UGGT^{FL}$ as an antigen (GeneFrontier Co., Ltd., Japan). For epitope mapping of the obtained anti-UGGT antibody, we prepared a series of $UGGT^{N}$-derived fragments. Based on the previous bioinformatic analysis[11], we prepared four fragments that were expressed as the soluble fraction in *E. coli*: Segment 1 (residues 672–833), Segment 2 (residues 469–833), Segment 3 (residues 29–833) and Segment 4 (residues 29–1189). The current crystallographic data revealed that Segments 1–4 contained the following domain fragments: Segment 1, Trx3 domain; Segment 2, Trx2 and Trx3 domains; Segment 3, Trx1, Trx4, Trx2, Trx3 and β-rich domains; and Segment 4, whole folding-sensor region. Dot blot analysis showed that the UGGT antibody reacted against Segments 3 and 4, but not Segments 1, 2 and CAT domain (Supplemental Fig. S11). These results together with the EM data (Fig. 4b) indicated that the UGGT antibody specifically recognises the Trx4 domain.

Table S1: Crystallographic data collection and refinement statistics for the UGGT folding-sensor domain

**Data collection**	$UGGT^N$
Crystallisation conditions*	2.00 M ammonium sulphate, 0.1 M Tris-HCl (pH 7.0), 10% IPTG, 5 days, 25 °C
Space group	$P3_212$
Unit cell parameters (*a*, *b*, *c* [Å])	195.1, 195.1, 142.3
Beamline	PF-AR NE3A
Wavelength (Å)	0.97864
Resolution range (Å)	50–3.10
Number of total reflections	285,343
Number of unique reflections	105,778
R_{merge} (%)	4.2 (63.9)
I / σ(I)	14.4 (1.4)
$CC_{1/2}$	0.999 (0.768)
Completeness (%)	96.4 (98.3)
Redundancy	2.7 (2.8)
**Refinement**	
Resolution range (Å)	19.97–3.10
R_{work}/R_{free} (%)	23.2/27.8
R.m.s.d. bond length (Å) and angles (°)	0.014/2.27
Ramachandran plot (%)	
Most favoured	74.2
Additionally allowed	22.7
Generously allowed	2.5
Disallowed	0.5
Number of atoms (A/B)	7159/7159
Average *B*-values (Å^2)	124.8/121.7

*Crystals were cryoprotected with the crystallisation buffer containing 15% glycerol and flash-cooled in liquid nitrogen.

Table S2: SAD and MAD data collection of the UGGT folding-sensor domain

**Data collection**	SeMet-non-mutated $UGGT^N$ (Peak)	K_2PtCl_4-$UGGT^N$ (Pt-edge)	K_2PtCl_4-$UGGT^N$ (Pt-high-remote)
Crystallisation condition*	1.95 M ammonium sulphate, 0.1 M Tris-HCl (pH 7.0), 8.3 mM *n*-decanoylsucrose, 5 days, 20 °C	[Crystallisation] 2.00 M ammonium sulphate, 0.1 M Tris-HCl (pH 7.0), 8.3% IPTG, 5 days, 25 °C [Soaking] 2.0 M ammonium sulphate, 0.1 M Tris-HCl (pH 7.0), 8.3% IPTG, 5 mM K_2PtCl_4, 6 h, 25 °C	Same as Pt-edge
Space group	$P6_222$	$P6_222$	$P6_222$
Unit cell parameters (*a*, *b*, *c* [Å])	196.4, 196.4, 143.9	195.4, 195.4, 142.6	195.1, 195.1, 142.7
Beamline	PF BL1A	SPring-8 BL44XU	SPring-8 BL44XU
Wavelength (Å)	0.97920	1.07227	1.05404
Resolution range (Å)	50–3.50	50–3.60	50–3.61
Number of total reflections	403,575	227,315	226,510
Number of unique reflections	21,052	34,727	34,466
R_{merge} (%)	14.5 (162.6)	9.2 (107.4)	7.6 (98.2)
$I / \sigma(I)$	22.1 (1.7)	13.2 (2.0)	14.5 (2.0)
$CC_{1/2}$	(0.892)	0.999 (0.678)	0.999 (0.690)
Completeness (%)	100.0 (100.0)	98.6 (99.0)	98.4 (97.8)
Redundancy	19.2 (20.5)	6.5 (6.5)	6.6 (6.6)

*Crystals were cryoprotected with the crystallisation buffer containing 15% glycerol and flash-cooled in liquid nitrogen.

Table S3: Crystallographic data collection of the domain-specific Met-labelled UGGT folding-sensor domain

**Data collection**	SeMet-<br>β-Trx1*-UGGT[N]	SeMet-<br>Trx2-3*-UGGT[N]	SeMet-<br>Trx4*-UGGT[N]
Crystallisation conditions*	1.95 M ammonium sulphate, 0.1 M Bis-tris-HCl (pH 6.5), 25 mM *n*-decanoylsucrose, 5 days, 20 °C	2.00 M ammonium sulphate, 0.1 M Tris-HCl (pH 7.0), 8.3% IPTG, 10 days, 20 °C	1.95 M ammonium sulphate, 0.1 M Tris-HCl (pH 7.0), 25 mM *n*-decanoylsucrose, 5 days, 20 °C
Space group	$P6_222$	$P6_222$	$P6_222$
Unit cell parameters (a, b, c [Å])	194.8, 194.8, 142.3	194.4, 194.4, 142.8	195.2, 195.2, 142.6
Beamline	SPring-8 BL44XU	NSRRC 13B1	SPring-8 BL44XU
Wavelength (Å)	0.97907	0.97934	0.97907
Resolution range (Å)	50–5.20	50–3.30	50–3.65
Number of total reflections	29998	688,203	217,974
Number of unique reflections	6552	24,516	18,458
R_{merge} (%)	20.0 (84.3)	13.3 (202.8)	10.4 (83.1)
I / σ(I)	14.4 (3.7)	43.3 (2.1)	38.3 (3.8)
$CC_{1/2}$	(0.773)	(0.838)	(0.845)
Completeness (%)	94.3 (95.0)	100.0 (100.0)	97.4 (99.1)
Redundancy	4.9 (5.0)	28.1 (27.3)	12.1 (12.2)

*Crystals were cryoprotected with the crystallisation buffer containing 15% glycerol and flash-cooled in liquid nitrogen.

Table S4: Crystallographic data collection and refinement statistics for the UGGT catalytic domain

**Data collection**	UDP-Glc-bound	UDP-bound
Space group	$P2_12_12_1$	$P2_12_12_1$
Unit cell parameters (*a*, *b*, *c* [Å])	45.5, 47.2, 129.8	
Beamline	PF-AR NW12A	PF BL5A
Wavelength (Å)	0.97934	1.00000
Resolution range (Å)	47.27–1.40 (1.48–1.40)	50–1.35 (1.43–1.35)
Number of total reflections	775,641	437,543
Number of unique reflections	56,063	62,275
R_{merge} (%)	14.0 (60.0)	8.8 (79.6)
$I / \sigma(I)$	7.7 (2.6)	12.8 (1.9)
$CC_{1/2}$	0.994 (0.834)	0.998 (0.750)
Completeness (%)	100.0 (100.0)	99.1 (94.5)
Redundancy	7.1 (7.0)	7.0 (6.2)
**Refinement**		
Resolution range (Å)	20.0–1.40	20.0–1.35
R_{work} / R_{free} (%)	14.2/17.4	16.8/20.8
R.m.s.d. bond length (Å) and angles (°)	0.016/1.59	0.014/1.67
Ramachandran plot (%)		
Most favoured	93.9	94.6
Additionally allowed	6.1	5.4
Generously allowed	0	0
Disallowed	0	0
Number of molecules		
Protein atoms	2305	2240
Ligands	43	34
Water molecules	263	220
Average *B*-values (Å^2)		
Protein atoms	17.3	15.4
Ligands	18.2	13.9
Water molecules	28.7	23.7

Table S5: SAXS data collection and refinement statistics for the full-length UGGT

**Data collection parameters**	$UGGT^{FL}$	$UGGT^{N}$
Diffractometer	Nano-Viewer (RIGAKU)	
Detector	PILATUS 200K	
Beam geometry	pin hole slit (ϕ0.8 mm)	
Wavelength (Å)	1.5418	
Q range	0.012–0.25	
Exposure time (min)	30	
Concentration range (mg/ml)	2–5	
**Structural parameters**		
I(0)/conc. (from *P*[*r*])	21.7 ± 0.2	18.9 ± 0.3
*R*g (Å) (from *P*[*r*])	45.2 ± 0.5	46.5 ± 0.8
I(0)/conc. (from Guinier)	21.3 ± 0.8	19.0 ± 0.5
*R*g (Å) (from Guinier)	45 ± 2.0	46 ± 1.2
D_{max} (Å)	150	155
Porod volume estimate ($Å^3$)	392000	393000
**Molecular mass determinant**		
M.W. (kDa) (from Guinier *I*[0])	164 ± 6.0	146 ± 2.3
Calculated M.W. (kDa)	170	123

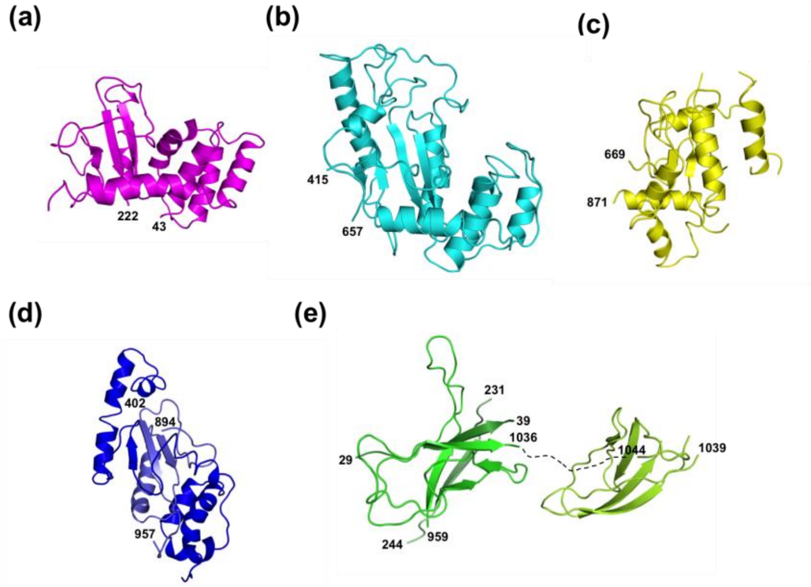

Fig. S1. Structures of individual domains of UGGT^{N}; (a) Trx1, (b) Trx2, (c) Trx3, (d) Trx4 and (e) β-rich domains. Positions of N- and C-terminal domain boundaries are indicated with the residue numbers. In the models of Trx4 and β-rich domains, the nonconsecutive segments are shown in same colors together with the residue numbers. N- and C-terminal subdomains of the β-rich domain are coloured green and lemon, respectively. Homology model of the C-terminal β-rich subdomain was constructed by SWISS-MODEL[31] using crystal structure of β-rich domain of human carboxypeptidase N (PDB code: 2NSM) as a template.

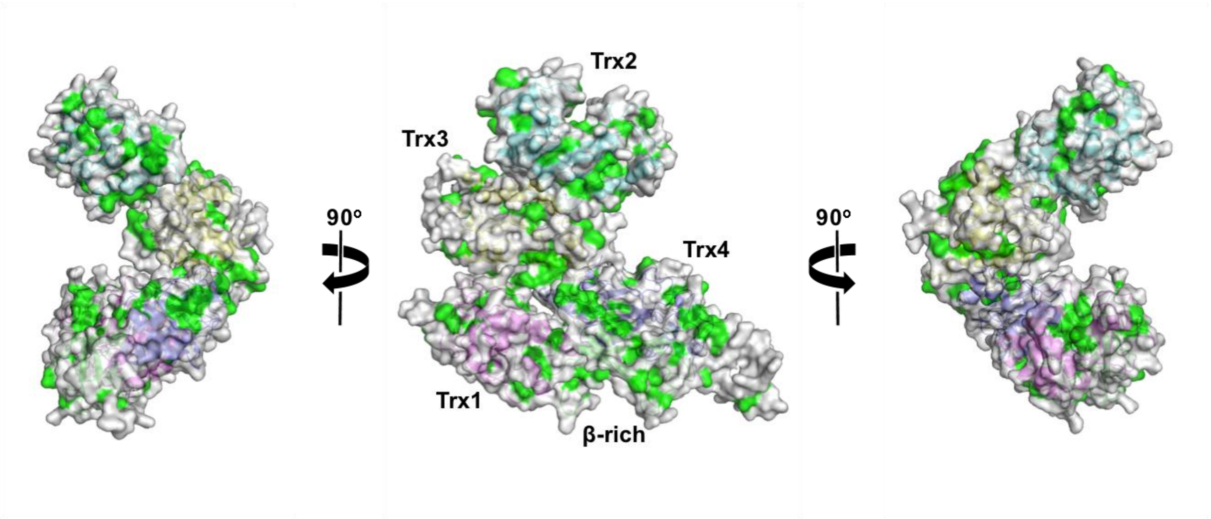

Fig. S2. Surface hydrophobicity of folding-sensor domain of UGGT. Molecular surface representation of the folding-sensor domain of UGGT. The hydrophobic residues are shown in green. The model was created by SWISS-MODEL[31] complementing with the disordered segments in the crystal structure.

```
                   1198 1199
Td      ADINIFSVASGHLYERMLNIMMVSVMKHTKHSVKFWFIEQFLSPSFKKFLPHLAKEYGFSYEMVTYKWPHWLRG
Sp      ASINIFSVASGHLYERFLYIMTKSVIEHTDKKVKFWFIENFLSPSFKSSIPAIAKKYNFEYEYITYNWPHWLRK
Dm      ETINIFSVASGHLYERLLRIMMVSLLKHTKSPVKFWFLKNYLSPQFTDFLPHMASEYNFQYELVQYKWPRWLHQ
Rn      DIINIFSVASGHLYERFLRIMMLSVLKNTKTPVKFWFLKNYLSPTFKEFIPYMAKKYNFQYELVQYKWPRWLHQ
Hs      DIINIFSVASGHLYERFLRIMMLSVLKNTKTPVKFWFLKNYLSPTFKEFIPYMANEYNFQYELVQYKWPRWLHQ
LgtC    --MDIVFAADDNYAAYLCVAAKSVEAAHPDTEIRFHVLDAGISEANRAAVAANLRGGGNIRFIDVNPEDFAGF

            1268 1272                      1294 1296                   1322
Td      QREKQREIWGYKILFLDVLFPLSLDKVIFVDADQIVRTDMYDLVQLDLEGAPYGFTPMCDSRKE---------M
Sp      QEEKQREIWGYKILFLDVLFPLELHKVIYVDADQIVRADLQELMDMDLHGAPYGYTPMCDSREE---------M
Dm      QTEKQRTIWGYKILFLDVLFPLNVRKIIFVDADAIVRTDIKELYDMDLGGAPYAYTPFCDSRKE---------M
Rn      QTEKQRIIWGYKILFLDVLFPLVVDKFLFVDADQIVRTDLKELRDFNLDGAPYGYTPFCDSRRE---------M
Hs      QTEKQRIIWGYKILFLDVLFPLVVDKFLFVDADQIVRTDLKELRDFNLDGAPYGYTPFCDSRRE---------M
LgtC    PLNIRHISITTYARLKLGEYIADCDKVLYLDIDVLVRDSLTPLWDTDLGDNWLGACIDLFVERQEGYKQKIGMA

          1329 1331  1339         1351 1352                           1386 1388
Td      EGFRFWKQGYWKSHLRGRPYH----ISALYVVDLNRFRALAAGDRLRGQYHTLSADPNSLANLDQDLPNNMQAM
Sp      EGFRFWKKGYWKKFLRGLKYH----ISALYVVDLDRFRKMGAGDLLRRQYQLLSADPNSLSNLDQDLPNHLQHL
Dm      EGFRFWKQGYWRSHLMGRRYH----ISALYVVDLKRFRKIAAGDRLRGQYQALSQDPNSLSNLDQDLPNNMIHQ
Rn      DGYRFWKSGYWASHLAGRKYH----ISALYVVDLKKFRKIAAGDRLRGQYQGLSQDPNSLSNLDQDLPNNMIHQ
Hs      DGYRFWKSGYWASHLAGRKYH----ISALYVVDLKKFRKIAAGDRLRGQYQGLSQDPNSLSNLDQDLPNNMIHQ
LgtC    DGEYYFNAGVLLINLKKWRRHDIFKMSCEWVEQYKDVMQYQDQDILNGLFKGGVCYANSRFNFMPTNYAFMANR

                     1339 1351 1352       1427 1429 1430    1438
Td      IPIKSLPQEWLWCETWCADEDL----KTARTIDLCNNPLTKEPKLDRARRQVPEWTEYDNEIAELAVRVRAAS-
Sp      IPIYSLPQDWLWCETWCSDESL----KTAKTIDLCQNPLTKEKKLDRARRQVSEWTSYDNEIASVLQTASSQS-
Dm      VAIKSLPDDWLWCQTWCSDSNF----KTAKVIDLCNNPQTKEAKLTAAQRIVPEWKDYDAELKTLMSRIEDHEN
Rn      VPIKSLPQEWLWCETWCDDASK----KRAKTIDLCNNPMTKEPKLEAAVRIVPEWQDYDQEIKQLQTLFQEEK-
Hs      VPIKSLPQEWLWCETWCDDASK----KRAKTIDLCNNPMTKEPKLEAAVRIVPEWQDYDQEIKQLQIRFQKEK-
LgtC    FASRHTDPLYRDRTNTVMPVAVSHYCGPAKPWHRDCTAWGAERFTELAGSLTTVPEEWRGKLAVPHRMFSTKR-

Td      ----------KQSRREEDEKKDV---------------------
Sp      ----------DKEFEEKDNNSSPDEL------------------
Dm      SHSRDSAVDDSVDDSVEVTTVTPSHEPKHGEL------------
Rn      --------ELGTLHEEETQEGSQKHEEL----------------
Hs      --------ETGALYKEKTKEPSREGPQKREEL------------
LgtC    --------MLQRWRRKLSARFLRKIY------------------
```

Fig. S3. Sequence alignment of the catalytic domain of UGGT across species (Td, *T. dupontii*; Sp, *Schizosaccharomyces pombe*; Dm, *Drosophila melanogaster*; Rn, *Rattus norvegicus*; Hs, *Homo sapiens*) together with *Neisseria meningitidis* α-galactosyltransferase, LgtC. The secondary structures of *T. dupontii* UGGT^{CAT} are indicated above the amino acid sequence. Residues involved in the ligand interactions are highlighted in yellow.

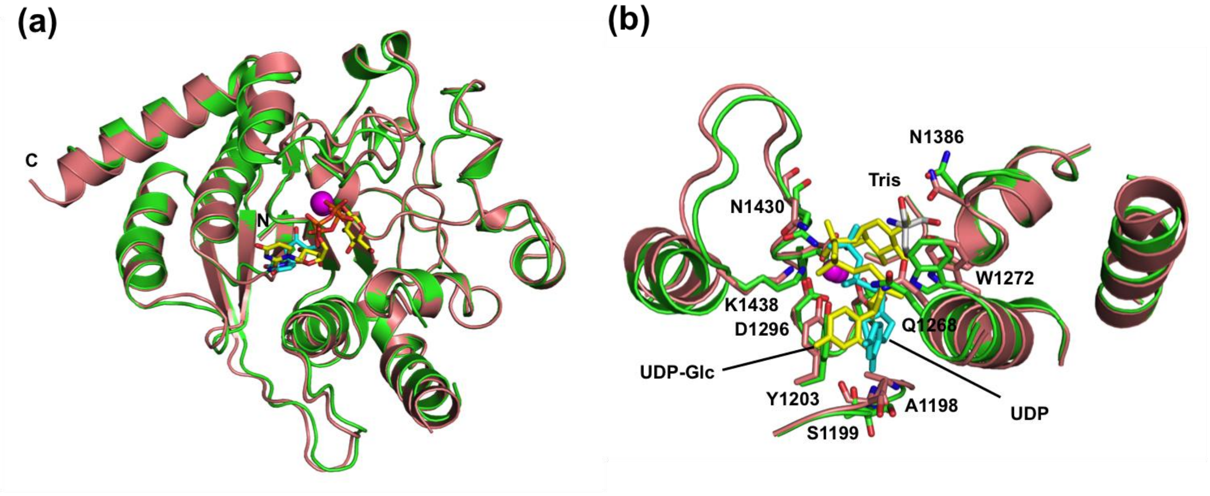

Fig. S4. Structural comparison between UDP-Glc- and UDP-bound structures of UGGT^{CAT}. The UDP-Glc-bound and UDP-bound forms are coloured pink and green, and the overall and close-up views are presented in (a) and (b), respectively. The bound Ca^{2+} is shown as a magenta sphere. The bound UDP-Glc, UDP and Tris molecules are shown in yellow, cyan and white, respectively. Residues involved in the ligand binding are shown in stick models.

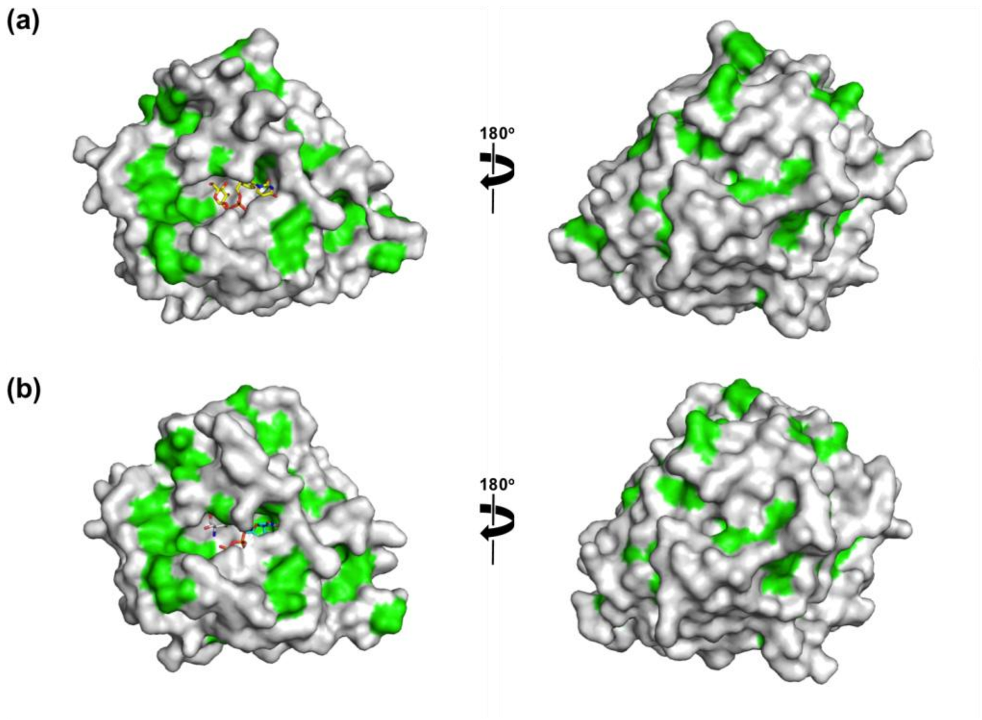

Fig. S5. Surface hydrophobicity of catalytic domain of UGGT. Molecular surface representation of the catalytic domain of UGGT: (a) UDP-Glc-bound form, (b) UDP-bound form. The hydrophobic residues are shown in green.

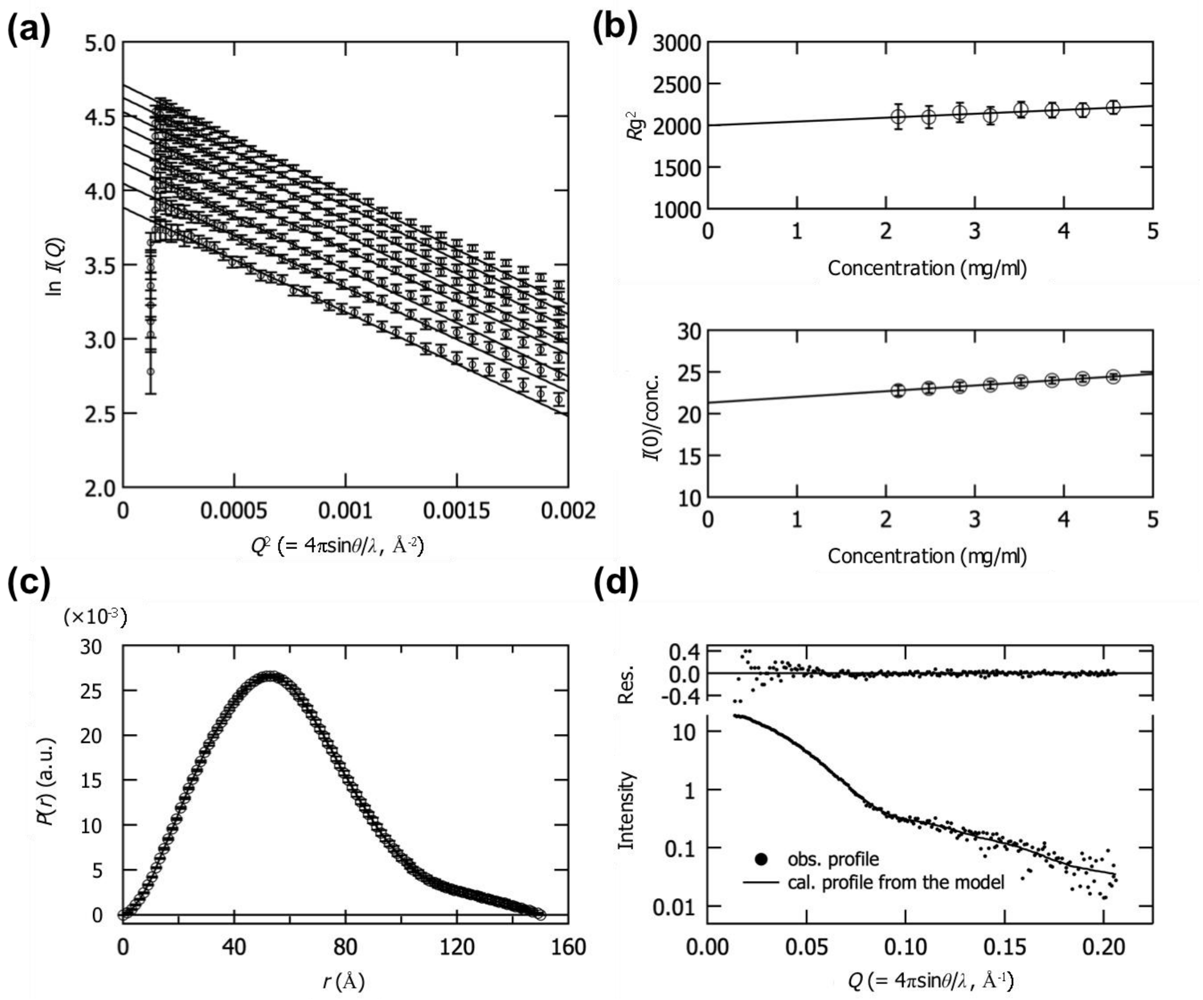

Fig. S6. (a) Guinier plots of UGGT^{FL} at different concentrations. (b) Concentration dependence of Rg^2 of UGGT (upper panel). Rg^2 was plotted against protein concentration. Concentration dependence of *I*(0)/conc. for UGGT (lower panel). (c) *P*(*r*) function of UGGT calculated from the SAXS curve extrapolated to a protein concentration of zero. (d) The calculated curve from the DAMMIN bead model superposed on the experimental scattering curve of the UGGT^{FL}.

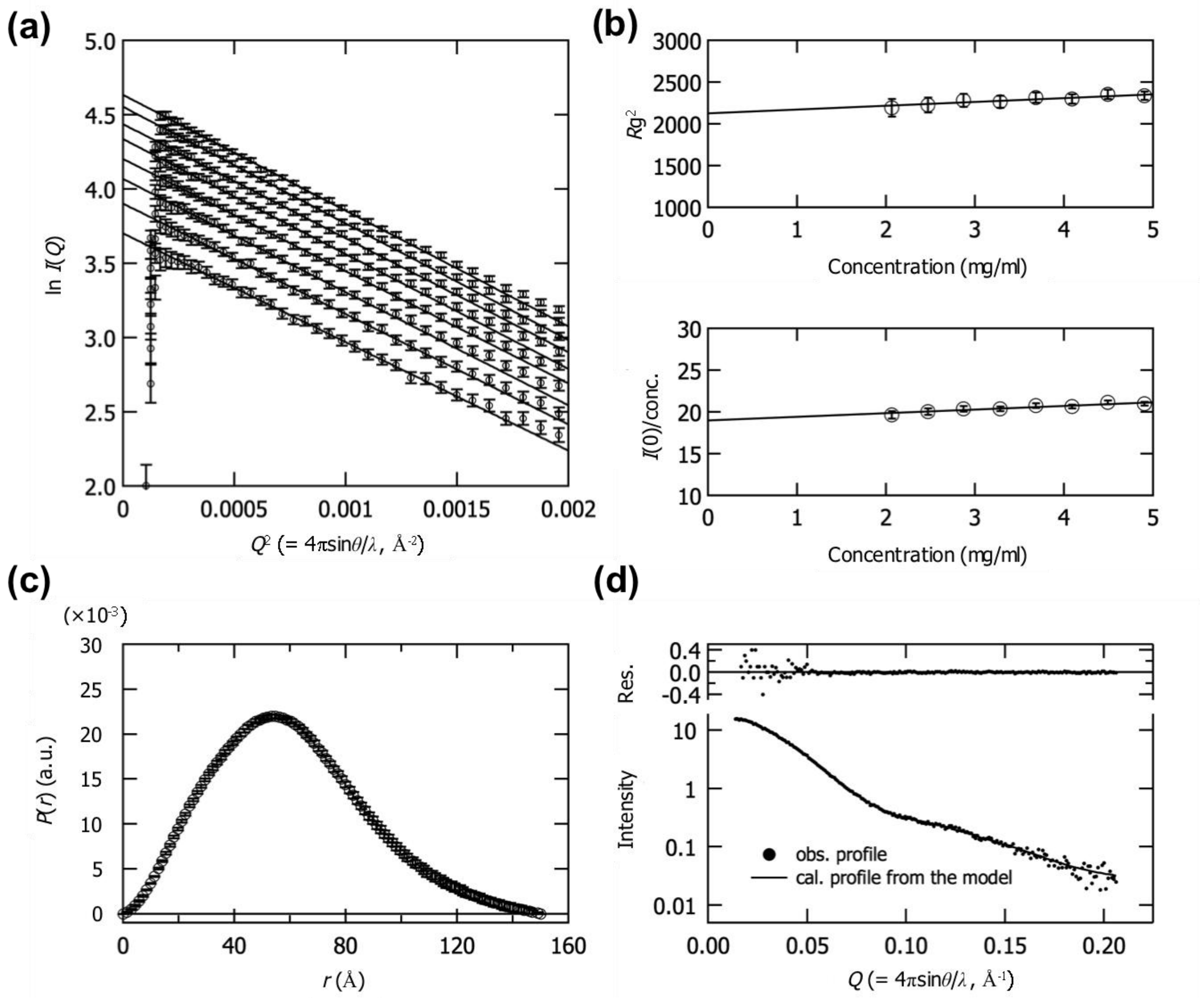

Fig. S7. (a) Guinier plots of UGGT^N at different concentrations. (b) Concentration dependence of Rg^2 of UGGT^N (upper panel). Rg^2 was plotted against protein concentration. Concentration dependence of I(0)/conc. for UGGT^N (lower panel). (c) P(r) function of UGGT^N calculated from the SAXS curve extrapolated to a protein concentration of zero. (d) The calculated curve from the DAMMIN bead model superposed on the experimental scattering curve of the UGGT^N.

29-1480<br>(FL)	Segment 1<br>672-833<br>(Trx3)	Segment 2<br>469-833<br>(Trx2-3)
Segment 3<br>29-833<br>(β+Trx1+Trx4+<br>Trx2+Trx3)	Segment 4<br>29-1189<br>(N)	1190-1480<br>(CAT)

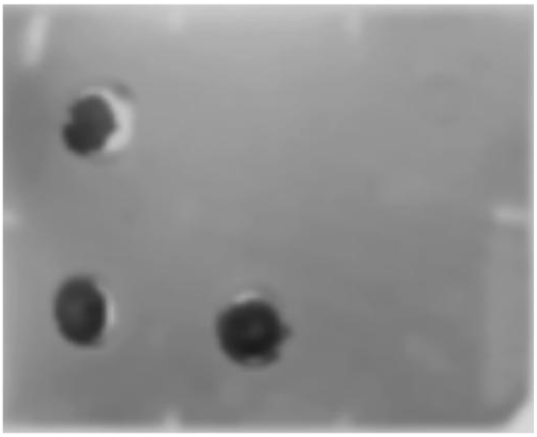

Fig. S8. Epitope mapping of anti-UGGT antibody. Epitope mapping of the antibody was performed by a dot blot method using the CAT domain and four fragments derived from UGGT^{N}.

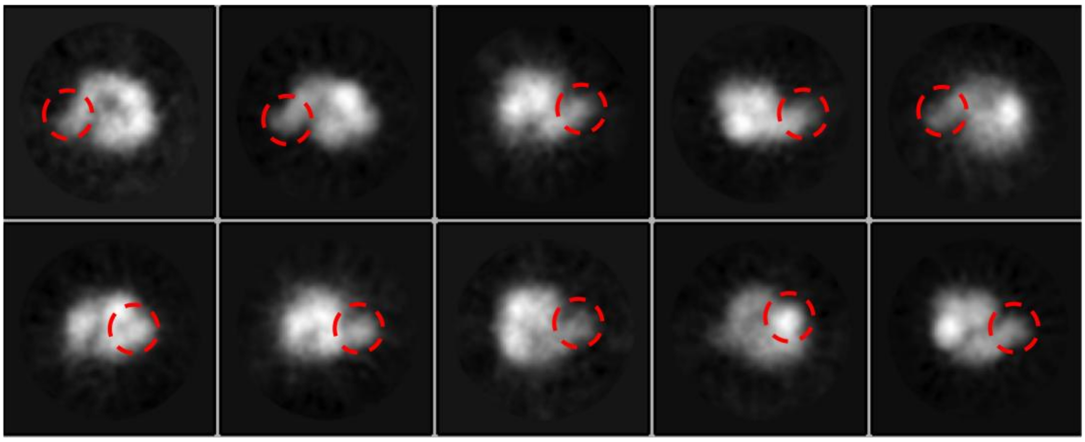

Fig. S9. Negative-stain EM analysis of UGGT^{FL}/anti-Trx4 Fab complex. 2D classes of UGGT particle subjected to single-particle negative-EM analysis. Predicted positions of anti-Trx4 Fab are marked with red dashed circles.

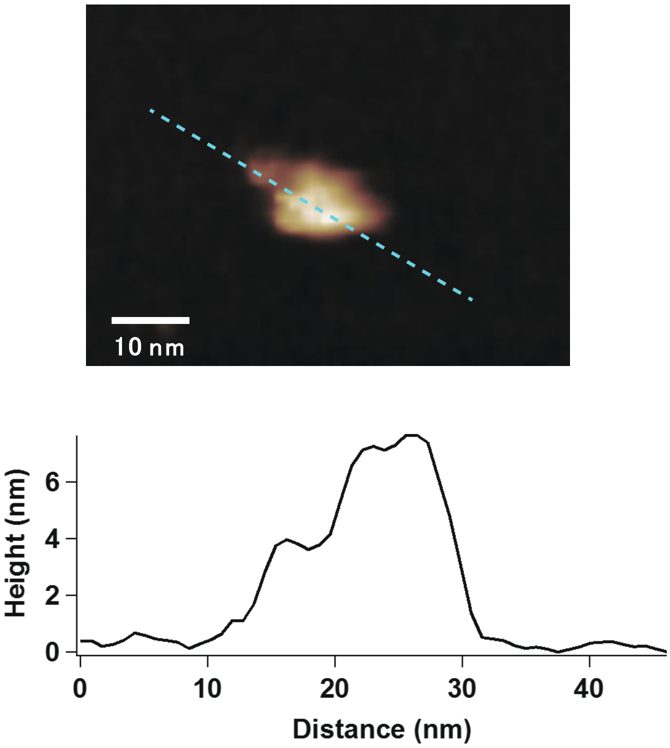

Fig. S10. Typical HS-AFM image of N-terminally His_6-tagged full-length UGGT (Upper, Sample #10, Supplemental Video S1) with a cross-sectional profile (lower).

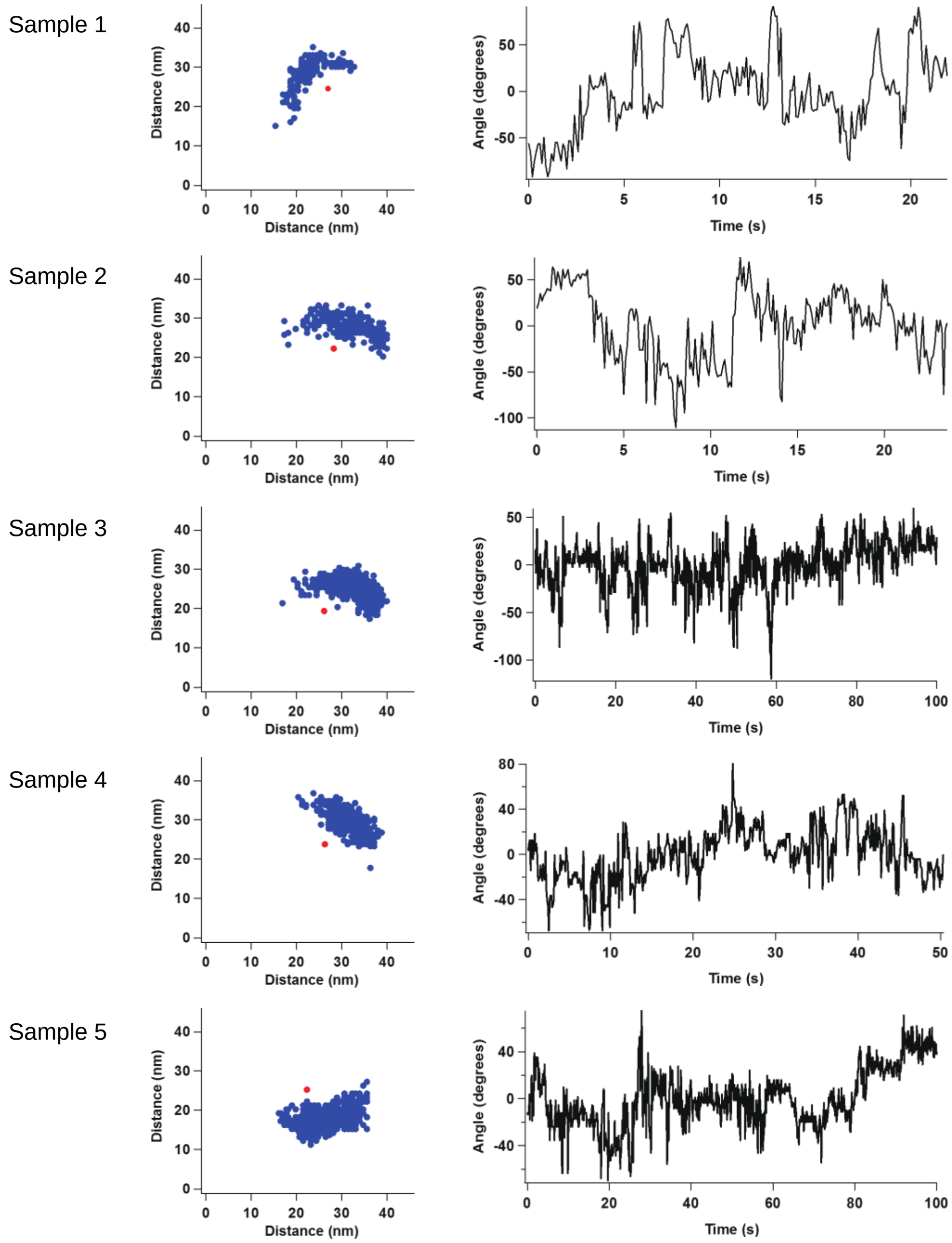
Sample 1
Sample 2
Sample 3
Sample 4
Sample 5
Distance (nm)
Distance (nm)
Angle (degrees)
Time (s)

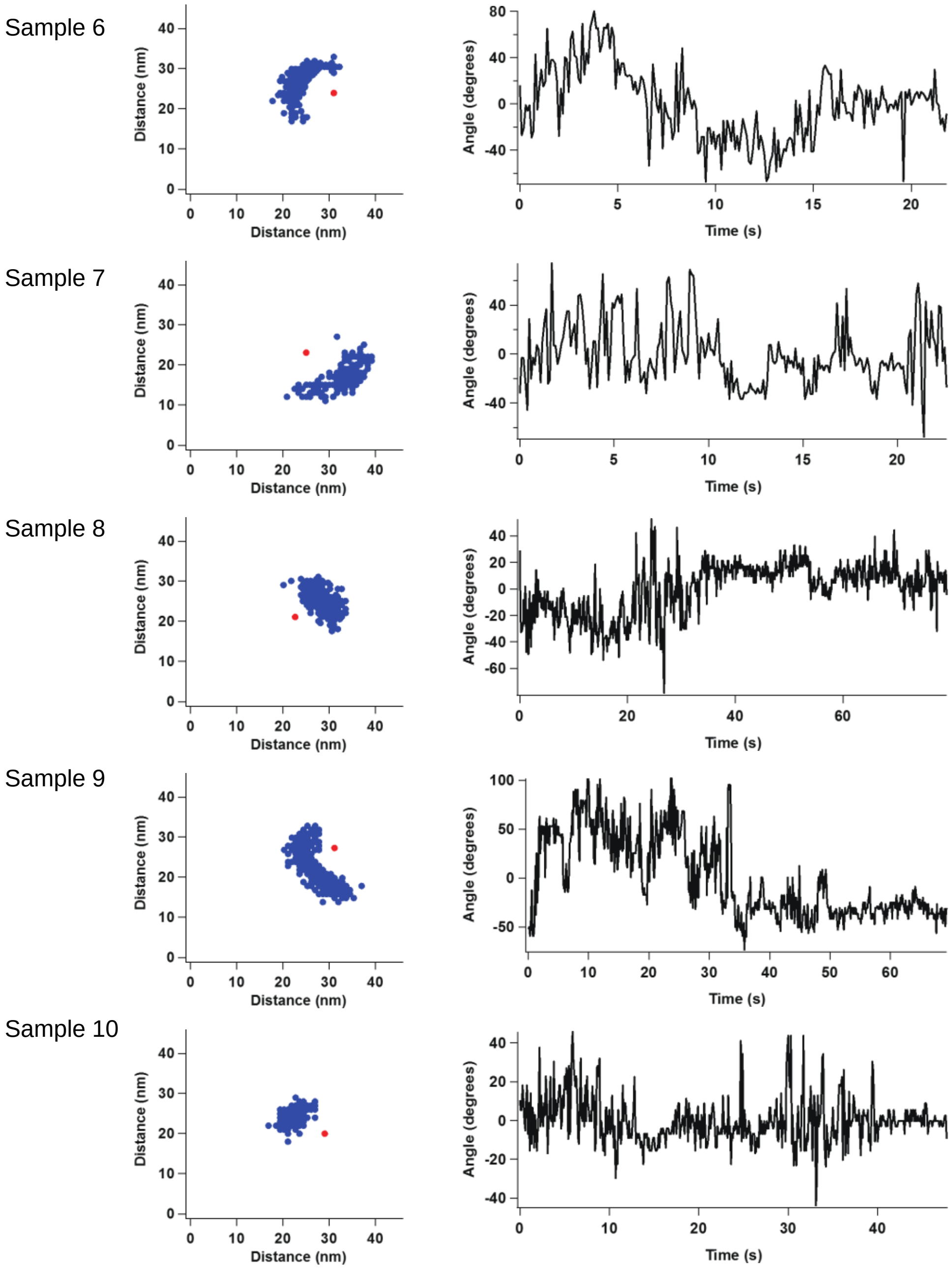

Fig. S11. Distribution of relative positions between centres of N- and C-lobes of *T. dupontii* UGGT in the HS-AFM measurements. The red plot indicates the normalised position of the centre of the N-lobe, whereas the blue plots indicate those of the C-lobe (left panel). Time series of the angles between two lobes (right panel). Zero angle corresponds to the averaged value in each dataset.

Video S1. Typical HS-AFM real-time image of N-terminally His_6-tagged, $UGGT^{FL}$ (Sample #10). The N-terminally His_6-tagged UGGT was applied to Ni^{2+}-treated AFM surface.

Video S2. Typical HS-AFM real-time image of C-terminally His_6-tagged, $UGGT^{FL}$. The C-terminally His_6-tagged UGGT was applied to the Ni^{2+}-treated AFM surface.

Video S3. Typical HS-AFM real-time image of deformed $UGGT^{FL}$ (N-terminally His_6-tagged construct). The deformed images were obtained by intentional disruption using an AFM tip.
